# A papilloma in a large cyst of the breast: A case report

**DOI:** 10.1016/j.ijscr.2018.11.013

**Published:** 2018-11-16

**Authors:** Hwan Soo Kim, Yang Hei Kim, Sung Bae Park

**Affiliations:** Department of Pediatric, Kangwon National University Hospital, Kangwon National University School of Medicine, Chuncheon, South Korea

**Keywords:** Intracystic lesions, Large cyst, Papilloma, Breast

## Abstract

**Introduction:**

Intracystic lesions of the breast prove to be benign more often than malignancy, most intracystic lesions are benign papillomas. Generally sizes of cysts that contain papilloma are small. Relatively cysts that contain papillary carcinoma are large. A papilloma in a large cyst of the Breast is rare.

**Presentation of case:**

We report a rare case of a papilloma in a large cyst of the breast that presented a painless palpable mass. A 53-yr-old woman noticed palpable mass in her right breast. Ultrasonography indicated a 7 × 7 cm sized large cyst. There is a 2 × 2.5 cm sized hyper-echoic, polypoid mass in the cyst. On fine-needle aspiration was not obtained malignant cells. Excisional biopsy was done. Pathologic finding was papilloma.

**Discussion:**

Tumors in a large cyst of the breast is difficult in distinguish benign from malignancy by preoperative imaging, cytological and histological study.

**Conclusion:**

Excisional biopsy should be performed before cancer surgery such as mastectomy to manage the cysts with intracystic growths.

## Highlights

• Intracystic lesions of the breast prove to be benign more often than malignancy.
• Most intracystic lesions are benign papillomas.
• Generally sizes of cysts that contain papilloma are small. Relatively cysts that contain papillary carcinoma are large.
• We present a 53-year-old with a papilloma in a large cyst of the breast that presented a painless palpable mass.
• Excisional biopsy may be needed to make a proper diagnosis for the suspicious lesion.

## 1 Introduction

Intracystic papillary lesions of the breast are rare, accounting for less than 1∼3% of all breast lesions, once identified as papillary, the categorization of the lesion as benign(papilloma), or malignancy(papillary carcinoma). Almost the lesions prove to benign more than malignancy [1,2]. The sizes of cysts that contain papilloma are smaller than papillary carcinoma, and intracystic papillary lesions smaller than 3 cm are generally benign [3,4]. Herein we report a rare case of a papilloma in a large cyst of the breast, and we review the relevant literatures.

The work in this case has been reported in line with the SCARE criteria [5].

## 2 Presentation of case

A 53-yr-old woman visited our hospital due to palpable mass in her right breast that had been rapid growing for approximately 2 months. The patient did not complain pain and had no nipple discharge. She had no specific family history and breast cancer risk factors. On physical examination, we palpated a well marginated, round, movable mass in right breast subareolar area.

**Fig. 1:**
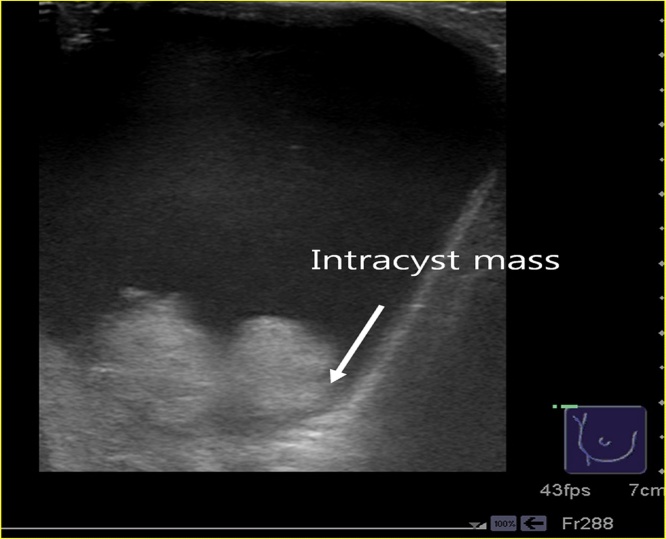
Ultrasonographic finding of the cyst. There is a 2 × 2.5 cm sized hyper-echoic, polypoid mass (arrow) in the huge cyst.

We ordered a check mammography for breast lesion. But patient was not able to test because of the pain during examination. Ultrasonography showed a 7 × 7 cm sized large cyst. There is a 2 × 2.5 cm sized hyper-echoic, polypoid mass in the cyst (Fig. 1). On fine-needle aspiration, yellow color no bloody fluid was obtained and cytological examination showed no cellular atypia.

**Fig. 2:**
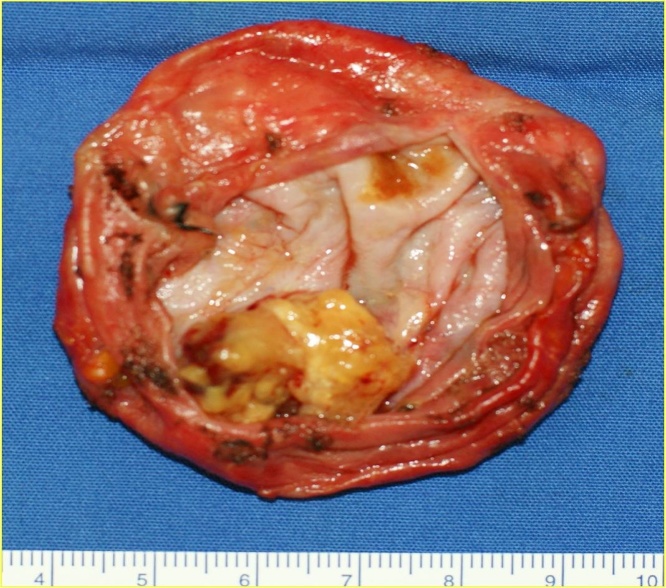
Gross finding of the cyst. Well circumscribed, large cyst with polypoid mass in the cyst.

**Fig. 3:**
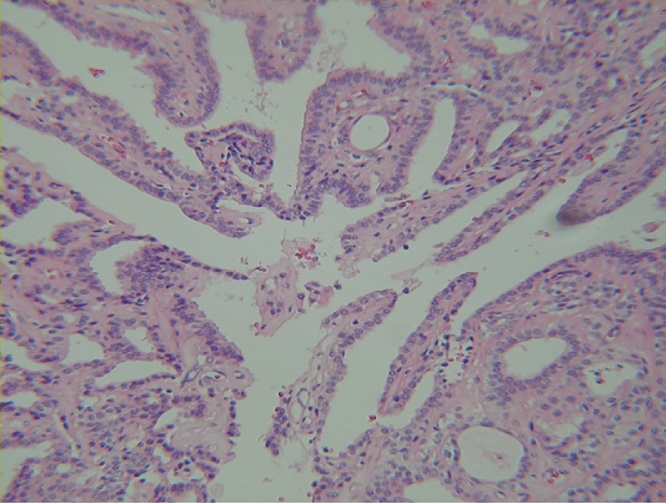
Microscopic finding of the mass. Intraductal proliferation of epithelial & myoepithelial cells overlying fibrovascular stalks(H & E staining, ×100).

We explained the result of examination to the patient as the cystic mass would be benign rather than malignancy, but malignancy was not ruled out. So we recommended surgical removal of the cystic mass, the patient agreed that. Excision biopsy was performed under general anesthesia. Amount of cystic fluid was 250cc. The resected specimen was a large cyst, measuring 7 × 7 cm and a intracystic papillary tumor, gray-brown color, measuring 2 × 2.5 cm, on the base of the cyst wall (Fig. 2). The histological diagnosis of intracystic papillary tumor was benign papilloma (Fig. 3).

## 3 Discussion

Intracystic breast lesions are associated with a variety of benign, atypical, and malignant pathologic diagnoses and the lesions prove to be benign more often than malignancy, Most intracystic lesions are benign papilloma [1,2,6].

The cyst develop by secretion and bleeding of an intraductal papilloma which distends and obstructs lactiferous ducts, causing cyst, and papilloma can be visualized inside the cyst [7]. Intracystic papillomas should be a disease distinguished from intracystic papillary carcinomas. In previous studies, the size of intracystic papillary lesions of the breast has been described as a differentiating clinical finding. That is to say, intracystic papillary lesions larger than 3 cm are almost malignancy [3,4].

Symptoms of intracystic papillomas are mainly painless, non-tender, palpable mass in subareolar area. Diagnosis is made by mammography, ultrasonography, computed tomography, magnetic resonance imaging and FNA or core needle biopsy. The mammographic finding of intracystic papilloma is non-specific, well marginated mass or negative on small lesion. Sonographically, it usually has a well circumscribed, septated cystic mass including irregular solid, hypo-echoic portion [8]. However, with these imaging modalities diagnosis of these lesions is difficult except in the case of intracystic carcinoma with invasive features on imaging. FNA cytology or core needle biopsy is important tools to get an accurate diagnose of intracystic papillomas or intracystic papillary carcinomas. Martorano [9] has stated that the cytological examination of the fluid obtained by puncture aspiration is not always enough: first because of the lack of epithelial cells in the liquid and second because of the presence of cellular atypia, which can also be visualized in the fluid of benign intracystic tumor.

There have been some reports that describe the methods to distinguish between intracystic papillomas and intracystic papillary carcinomas preoperatively [[10], [11], [12], [13]]. Shah [10] reported that immunohistochemistry increases the accuracy of diagnosis of benign papillary lesions in breast core needle biopsy specimens. Immunohistochemistry using myoepithelial markers, such as calponin and p63, has been shown to be useful for differentiating benign papilloma from intraductal papillary carcinoma as myoepithelial cells are readily demonstrable in the benign papillomas than intraductal papillary carcinomas. However, Tsuda [11] reported that examination of loss of heterozygosity on should be helpful for differential diagnosis of intracystic papillary tumor, since papillomas showed no LOH. In the present studies, fiberotic ductoscopy or percutaneous endoscopic procedures are available for the evaluation of intracystic breast lesions [12,13]. In patients with an intracystic mass, reliable differentiation is not possible with any imaging modality, and fine-needle aspiration and cytological examination rarely lead to a definitive diagnosis between benign papilloma and papillary carcinoma. Some studies reported that intracystic papillary lesions are mostly diagnosed with core needle biopsy [14]. However, it cannot always differentiate from benign and malignancy [[15], [16], [17]]. As a result, an excisional biopsy may be needed to make a proper diagnosis for the suspicious lesion [18,19].

## 4 Conclusion

A papilloma in a large cyst of the breast very rarely occurs. Although the size of the cysts with intracystic growths is large, it may be a benign tumor. Therefore, excisional biopsy should be performed before cancer surgery such as mastectomy to manage the cysts with intracystic growths.

## Conflicts of interest

No potential conflict of interest relevant to this article was reported.

## Sources of funding

None.

## Ethical approval

Not applicable. The study is exempt from ethical approval in our institution.

## Consent

Written informed consent was obtained from the patient for publication of this case report and accompanying figures.

## Author contribution

Kim Hwan Soo and Yang Hei Kim- Data collection, writing the paper.

Sung Bae Park - study concept, writing the paper, advised and designed the report.

## Registration of research studies

N/A.

## Guarantor

Sung Bae Park.

## Provenance and peer review

Not commissioned, externally peer reviewed.
